# The Application of Gene Expression Profiling in Predictions of Occult Lymph Node Metastasis in Colorectal Cancer Patients

**DOI:** 10.3390/biomedicines6010027

**Published:** 2018-03-02

**Authors:** Noshad Peyravian, Pegah Larki, Ehsan Gharib, Ehsan Nazemalhosseini-Mojarad, Fakhrosadate Anaraki, Chris Young, James McClellan, Maziar Ashrafian Bonab, Hamid Asadzadeh-Aghdaei, Mohammad Reza Zali

**Affiliations:** 1 Basic and Molecular Epidemiology of Gastrointestinal Disorders Research Center, Research Institute for Gastroenterology and Live Diseases, Shahid Beheshti University of Medical Sciences, Tehran 19839-63113, Iran; n.peyravian@gmail.com (N.P.); dr.larki@gmail.com (P.L.); ehsangharib55@gmail.com (E.G.); hamid.asadzadeh@sbmu.ac.ir (H.A.-A.); 2 Gastroenterology and Liver Diseases Research Center, Research Institute for Gastroenterology and Liver Diseases, Shahid Beheshti University of Medical Sciences, Tehran 19839-63113, Iran; mrzali222@gmail.com; 3 Colorectal Division of Department of Surgery, Taleghani Hospital, Shahid Beheshti University of Medical Sciences, Tehran 19839-63113b, Iran; dr.anaraki47@gmail.com; 4 School of Allied Health Sciences, Faculty of Health and Life Sciences, De Montfort University, Leicester LE1 9BH, UK; chris.young@dmu.ac.uk; 5 School of Biological Sciences, University of Portsmouth, Portsmouth PO1 2UP, UK; chopsalotapepl@yahoo.co.uk; 6 Department of Biological Sciences, University of Chester, Chester CH1 4BJ, UK

**Keywords:** gene expression profiling (GEP), lymph node metastasis (LNM), colorectal cancer (CRC)

## Abstract

A key factor in determining the likely outcome for a patient with colorectal cancer is whether or not the tumour has metastasised to the lymph nodes—information which is also important in assessing any possibilities of lymph node resection so as to improve survival. In this review we perform a wide-range assessment of literature relating to recent developments in gene expression profiling (GEP) of the primary tumour, to determine their utility in assessing node status. A set of characteristic genes seems to be involved in the prediction of lymph node metastasis (LNM) in colorectal patients. Hence, GEP is applicable in personalised/individualised/tailored therapies and provides insights into developing novel therapeutic targets. Not only is GEP useful in prediction of LNM, but it also allows classification based on differences such as sample size, target gene expression, and examination method.

## 1. Introduction

Colorectal cancer (CRC) is one of the most common causes of cancer-related deaths worldwide [1]. Currently, CRC patients are classified by staging of the tumours with the tumour/node/metastases (TNM) system validated by the American Joint Committee on Cancer (AJCC). In this system, patients are divided into various groups and subgroups, according to the size and extension of the primary tumour, its lymphatic involvement, and any metastatic features. Usually, advanced cancer stages are characterised by metastases to local lymph nodes (LNs) or other organs, which lead to poor prognosis [2]. Among the criteria included in the TNM system, LNs are the strongest predictive/prognostic marker for evaluating patient outcomes and choosing the best therapeutic strategy [3,4].

Curative surgery improves the CRC patient’s status at least in early stage, but the healing process is not adequate by itself [5]. For example, five-year survival rates published by the American Cancer Society (ACS) are highly variable: 74% for stage I, 67% for stage IIA, 59% for stage IIB, 37% for stage IIC, 73% for stage IIIA, 46% for stage IIIB, 28% for stage IIIC, and 6% for stage IV disease (https://www.verywell.com, Updated 9 April 2017). In another report, nearly 30% of CRC cases with no record of their lymph node metastasis (LNM) status were found during the five-year follow-up to have died because of tumour recurrence. The authors of the latter study concluded that the presence of the occult lymph node metastases (micrometastases) that were not detected by routine examinations might be the cause [6].

The prevalence of micrometastases in patients with early CRC has recently been widely investigated. Published data indicate that even patients with stage I, who seem to have localised disease, may harbor micrometastases that were missed during the clinicopathological tests [7]. In line with this finding, Bonetti et al. (2011) showed that the occurrence of micrometastases was the main cause of death in patients with early-stage cancer [8]. Therefore, LN involvement has to be considered as a key factor in overall survival (OS) and disease-free survival (DFS). Thus, it may be also necessary to use molecular techniques to enhance this evaluation. In this paper, the role of gene expression profiling (GEP) in predictions of occult lymph node metastasis in colorectal cancer is reviewed.

## 2. Pathogenesis of CRC

Like other cancer types, CRC occurs by multiple misregulation of oncoproteins or tumour suppressors that impair the intra- and extra-cellular signal balances. The two most applicable models in this field are as follows:

(1) A model that consists of three molecular subtypes including:
  (a) chromosomal instability (CIN) [9],
  (b) CpG island methylator phenotype (CIMP) [10],
  (c) microsatellite instability (MSI) [11].
(2) A model of four consensus molecular subtypes (CMSs). The members of this model show the following discriminating features:
  (a) CMS1 (14%), microsatellite instability (MSI) and immune hyperactivation,
  (b) CMS2 (37%), epithelial involvement, wingless-type MMTV integration site family member (WNT) and MYC pathway interaction,
  (c) CMS3 (13%), epithelial and metabolic involvement,
  (d) CMS4 (23%), invasive and metastatic activation of transforming growth factor–β (TGF-β) [12].

These models facilitate the identification of germline mutations of the genes involved in DNA mismatch repair (MMR) including MutS protein homolog 2 and 6 (MSH 2,6), MutL protein homolog 1 (MLH1), and PMS1 homolog 2 (PMS2), which all cause MSI in patients with hereditary HNPCC/Lynch syndrome [13]. As in Model 1, the CIN approach help us to characterise the 85% of CRC cases with enhanced chromosomal gains (1q, 7p-q, 8q, 13q and 20p-q) and losses (8q21-pter, 15q11-15, 17p12-13, 18q12-21) [14,15,16]. In addition, 12 subsets of mutated genes have been identified that can trigger the cancer initiation [17]. Among them, the adenomatous polyposis coli (APC) mutation is the most frequent initiating event during the cell transformation. APC mutation by dysplastic aberrant crypt foci results in Wnt/β-catenin signaling pathway activation [18]. The subsequent changes may occur in Kirsten RAS (K-RAS) and tumour suppressor p53 protein. For example, the transition of colonic polyps into the tumour process is boosted by mutated K-RAS or p53 [19,20]. Besides the Wnt signaling cascade, TGF-β signaling intermediates including Sma and Mad-related protein 2 and 4 (SMAD 2,4), Runt-related transcription factor 3 (RUNX3), and Thrombospondin 1 (TSP1) are highly misregulated during CRC [21].

## 3. Gene Expression Profiling

As previously mentioned, DNA mutations have a huge impact on prognosis and survival rates. However, the impact of thousands of detected mutations on cancer progression has not yet been elucidated, and we are not sure whether some of these mutations cause the cancer (driver mutations) or emerge because of the cancer (passenger mutations). Currently, gene expression profiling of the primary tumours has received a great deal of attention due to its ability to create a detailed picture of the genetic and epigenetic alteration status of tumours.

What is the actual value of the gene expression signatures for the prediction of key events such as recurrence or LNM in CRC patients? This question has been the subject of numerous previous studies (Table 1 and Supplementary Material). Generally, these studies have shown that GEP is capable of evaluating the expression of numerous genes in a single test, thereby allowing a highly accurate depiction of cell function and status. The obtained expression pattern may be used to classify cells based on their function, type, or response to specific reagents [20].

The detection of high-risk patients with recurrent CRC is arguably the main challenge. In this regard, applying molecular assessment tools could be helpful in identifying cases of occult micrometastasis and potential suitability of adjuvant therapy [40,41]. Accordingly, new molecular approaches are required for rapid and accurate detection of occult lymph node metastasis.

Over the last decade, gene expression profiling by microarray has been a pioneering method in the detection of disease-related molecules. Compared with other standard techniques such as RT-PCR, which is not able to evaluate a large number of targets at once, microarray screening provides fast and reliable data with high accuracy from various samples. With microarray, it is now possible to analyse the whole expression pattern of a human genome within 48–72 h, thus gathering precious details about related molecular subtypes [42].

Using cDNA microarray technology, Kwon et al. (2004) examined and compared the expression profiling of 4608 genes in 12 CRC tumours versus in noncancerous tissues [31]. According to their report, 120 genes that regulate cell signalling, metabolism, proliferation, and apoptosis were expressed differently between the experimental groups.

Wang et al. (2004) tried to identify new prognostic markers for cancer relapse by DNA chip technology. Their achievement was a 23-gene panel allowing prediction of recurrence in Dukes’ B patients. The panel also provided insight regarding the underlying biological mechanism of rapid metastases; some of these genes are involved with tumour development and cell proliferation. For example, Tyrosine 3 mono-oxygenase tryptophan 5-monooxygenase activation proteins (YWHAH) and Regulator of chromosome condensation 1 (RCC1) are the most important genes governing the G2 checkpoint of the cell cycle and chromosome condensation initiation, respectively [27]. In the same year, Bertucci et al. (2004) compared 50 cancerous and noncancerous colonic tissues using a DNA microarray consisting of ~8000 spotted human cDNAs, in the process discovering that Guanine nucleotide binding protein subunit beta2 like-1 (GNB2L1), also named as RACK1, was the top-ranked gene overexpressed in cancer samples [23]. The product of this gene is a homologue of the beta subunit of G proteins, and participates in signal transduction and Protein Kinase C (PKC) activation. They also evaluated the Nucleoside diphosphate kinase A (NM23) level and noted that in NM23-positive patients, the chance of metastasis and death is significantly less than in the NM23-negative cases [23].

Arango et al. (2005) proved that the downregulation of Ras homolog gene, a small GTPase protein known to regulate the actin cytoskeleton, was correlated with shorter survival. This approach is not only useful in Dukes’ C patients’ recurrence predictions but also can be used in the design of clinical management algorithms [22].

Meeh and colleagues used digital long serial analysis of gene expression to elucidate the differences between node-negative and node-positive colorectal tumours. They reported that the development of node-positive CRC occurs, in part, through elevated levels of epithelial Fibronectin 1 (FN1). They suggested that the progression of the CRC from node-negative to node-positive disease may be facilitated partially by FN1 deregulation and the subsequent enhancement of tumour cell migration [29].

In 2009, Watanabe and colleagues determined that gene expression programming could be a useful tool in predicting recurrence in stage III colorectal cancer, and also identified calcineurin binding protein 1 (CABIN1) among discriminating genes that may play a key role in the development of recurrence [26]. Additionally, they identified 73 novel genes and transcripts the expression of which varied significantly between patients with or without LNM. Of these, 37 genes were upregulated and 36 showed lower expressions in cases with LNM compared with patients without LNM. The list of genes included tumour suppressor genes (ST7, BAP1) and transmembrane glycoprotein related to lymph node metastasis in prostate cancer (PSMA) [25].

The other family involved in cancer metastasis is the Forkhead box proteins (FOX). For example, FOXC2, also known as Forkhead Box C2 (FKHL14), has been found to be significantly elevated in patients with lymph node involvement and correlated with the degree of LNM [32]. Upregulation of the other member of this family, FOXP3, is linked with depression of the immune response by the accelerating the secretion of factors like TGF-β and Interleukin-10 [43].

In 2011, Salazar and colleagues developed a robust gene expression classifier (ColoPrint) that can predict relapses in patients with early-stage CRC. According to their study, this method can identify patients with stage II CRC who will experience a recurrence within five years after surgery [28]. The technique is not only able to predict the development of distant metastasis but also helps to identify the individuals who may be safely managed without chemotherapy independent of the clinical variables [30,44].

Besides ColoPrint, Lenehan et al. (2012) have developed a molecular prognostic examination able to identify tumour recurrence within three years in CRC cases having curative surgery. From analysing the expression changes of 18 key genes involved in regulation of cell signal transduction, gene expression, invasion, growth, angiogenesis, apoptosis, and antioxidation, they identified five genes that could be used in the prediction of tumour recurrence in CRC patients: BMI-1 polycomb ring finger oncogene (BMI), Vascular endothelial growth factor A (VEGFA), Ribosomal protein S10 (RPS10), Ets variant 6 (ETV6), and H3 histone, family 3B (H3F3B). However, the test was validated in stage I and II patients and the authors concluded that ≥12 lymph node samples would be required for accurate prognostication [30].

The results of another study on 196 genes in CRC patients determined that use of the expression pattern of main genes such as Annexin A3 (ANXA3), C-type lectin domain family 4 member M (CLEC4D), Lamin B (LMNB1), proline rich and Gla domain 4 (PRRG4), TNF alpha induced protein 6 (TNFAIP6), Lamin B1 (TNFAIP6), Vanin 1 (VNN1), and Interleukin 2 receptor subunit beta (IL2RB) that participate in tumour initiation and development could act as novel biomarkers for early detection of CRC [35]. In line with these findings, Ganepola (2014) proposed that gene expression signatures of Oncotype DX and ColoPrint could be good tools for management of early-stage colon cancer [45].

GEP has been investigated for its potential to predict the outcome of patients in other cancers, too. Méndez et al. (2011) identified five genes (Receptor accessory protein (1REEP1), Ring finger protein 145 (RNF145), CTONG2002744, Myosin VA (MYO5A), and FBXO32) that were differentially expressed between node-positive and node-negative oral squamous cell carcinomas (OSCC), and suggested that this model is applicable for identification of occult metastasis in patients [46]. Similarly, studies on primary lung adenocarcinomas, pancreatic, breast, bladder, and prostate cancers models depicted a marked alteration in gene expression patterns along with a high relative risk of nodal involvement [47,48,49,50,51]. 4. The Limitations of the GEP Approach

As already discussed, the GEP platform provides a unique opportunity to examine tens of thousands of different candidate genes at a given time. However, like other laboratory methods, GEP technology is limited. The data obtained from GEP are very simple and only consist of the candidate genes. GEP is not able to detect the interactions or signaling crosstalks. Therefore, other data analyses must be performed to elucidate the underlined network, for example, behind the antitumour drug resistance. Thus, different perceptions may be derived from a single raw data point. The other limitation of the GEP approach is the sample type and the method of preparation. The DNA of different types of cells and tissues of the body undergo multiple modifications and express various genes; hence, the quality of DNA extraction and isolation is directly related to the GEP output. This noise is usually resolved by increasing the sample size, but in particular cases, other alternatives such as RNA sequencing technology [52,53] or single-cell RNA sequencing (scRNA-Seq) [44] should be considered, subsequently. 

## 5. Conclusions

Overall, for prediction of tumour recurrence and metastasis, GEP analysis has marked advantages compared to routine clinical exams. Using microarray technology, various genes are identified that allow prediction of LNM in CRC cases. Based on this approach, a new classification of CRC has been introduced that reflects the different biological pathways and distinct prognostic features, allowing preselection of patients who would benefit from adjuvant therapies. However, more investigations are also needed to identify the genes associated with poor prognosis profiles, since these may actually prove interesting potential targets for rational development of new cancer drugs.

## Figures and Tables

**Table 1:** Summary of published reports on gene expression profiling (GEP) in colorectal cancer (CRC) patients during 2004–2018.

References	Samples/Method	Panel	Conclusion
Arango et al. (2005) [22]	137 fresh-frozen tumour Stage III CRC/Microarray analysis	22,283 probe sets	GEP predict recurrence in Dukes’ C
Bertucci et al. (2004) [23]	50 cancerous and noncancerous colon tissues/Microarray analysis	The panel of ~8000 genes (spotted human cDNA)	GEP can improve the prognostic markers
Watanabe et al. (2011) [24]	141 CRC patients Microarray analysis	40 discriminating probes	18 genes found to decrease in patients with lymph node metastasis (LNM) in comparison to those without metastases
Watanabe et al. (2009a) [25]	89 CRC Patients/Human U133 Plus 2.0 GeneChip^®^	73 novel discriminating genes	GEP may be useful in predicting the presence of LNM
Watanabe et al. (2009b) [26]	36 stage III CRC patients/Human U133 Plus 2.0 GeneChip^®^	The genes that are predictive for the presence of lymph node metastasis	GEP is useful in predicting recurrence in stage III colorectal cancer
Wang et al. (2004) [27]	74 patients with Dukes’ B CRC/Microarray U133a GeneChip^®^	Containing a total of 22,000 probe sets	A 23-gene signature that predicts recurrence in Dukes’ B patients
Salazar et al. (2010) [28]	188 fresh-frozen tumour with stage I to IV CRC/Agilent 44 K oligonucleotide arrays	-	Coloprint can distinguish low- and high-risk patients 18 genes
Meeh et al. (2009) [29]	25 fresh-frozen CRC tumour/Digital long serial analysis of gene expression	Sequenced to a depth of 26,060 unique tags	Development of LN in CRC occurs in part through elevated epithelial FN1 expression
Lenehan et al. (2012) [30]	74 CRC patients (FFPE)/TaqMan Low-Density Arrays	225 prespecified tumour genes	Onco-Defender-CRC capable of differentiating between patients at ‘‘high risk’’ from those at ‘‘low risk’’
Kwon et al. (2004) [31]	12 fresh-frozen CRC tumour/Microarray analysis	408 genes	GEP can predict LNM
Marisa et al. (2013) [32]	750 fresh-frozen CRC samples/Human U133 Plus 2.0 eneChip^®^	6 subtypes (Each contains 1000 genes)	GEP makes it possible to classify CRC samples based on genetic signatures and identify the targets for therapeutic attempts
Becht et al. (2016) [33]	1388 CRC tumour samples/Microarrays analysis	-	GEP is applicable in immune and stromal classification of CRC tumours
Inoue et al. (2015) [34]	One hundred FFPE tissue Samples/Microarrays analysis	-	GEP could explain the heterogeneity of unresectable advanced or recurrent CRC
Vishnubalaji et al. (2015) [35]	13 fresh-frozen consecutive sporadic CRCs matched with their adjacent normal mucosa/microarray chip and miRNA microarray chip	Genes involved in pathways of cell cycle, integrated cancer	The data revealed several hundred potential miRNA-mRNA regulatory networks in CRC and suggest targeting relevant networks as potential therapeutic strategy for CRC
Yamada et al. (2018) [36]	278 colorectal tissue samples/Real-time RT-PCR, cell culture, and RNA	Panel of lnc-RNAs	The data highlight the capability of RNA-seq to discover novel lncRNAs involved in human carcinogenesis, which may serve as alternative biomarkers and/or molecular treatment targets
Nguyen et al. (2015) [37]	The 1358 unique patients of six different CRC data sets/Microarray analysis	Panel of *CRC-113* gene signature	CRC-113 gene signature provides new possibilities for improving prognostic models and personalised therapeutic strategies
Gao et al. (2015) [38]	1005 patients with stage II CRC/Microarray analysis	Eight cancer hallmark–based gene signatures were identified to construct CSS (cancer-specific survival) (cancer-specific survival) sets for determining prognosis	The prediction accuracy for low-and high-risk disease significantly outperformed other gene signatures such as Oncotype DX and ColoPrint
Li et al. (2017) [39]	11 primary colorectal tumours/Single-cell RNA-Seq Method	Panel of 292 genes	Results demonstrate that unbiased single-cell RNA-Seq profiling of tumour and matched normal samples enables us to characterise aberrant cell states within a tumour

## Supplementary Materials

The following are available online at http://www.mdpi.com/2227-9059/6/1/27/s1.
